# Annotations

**Published:** 1903-08-29

**Authors:** 


### Annotations.

### Medicine or Surgery?

The alterations that are constantly going on in the relation between medicine and surgery are very curious. At what period a differentiation was first established between the two is not certain, but it is probable that in the early days of Western civilisation surgery was hardly regarded as a branch of the healing art at all. The field of possible surgery was so limited that nothing beyond plain common sense was needed to cover all its boundaries, every old soldier who could bind a broken limb or apply a balsam to a cut or sore being as good a surgeon as the best. Later on, the men of medicine and mystic learning included surgery in their accomplishments, but for centuries after this compliment had been paid to the art, those who practised it alone were of such low esteem, that without the sanction of the physician, though a limb might be set or a simple applied, the barber-surgeon could scarcely venture to whet, still less to use, his knife. Eventually the surgeons got a footing of their own and obtained a better social repute. "He did more to make us gentlemen than any other man," said a contemporary speaking of John Hunter, but for a long period after this the line between medicine and surgery was very clear and distinct. Of late years, however, the field of surgery has steadily broadened, and nearly every year now witnesses the invasion by surgery of some region which medicine had up to then imagined its own undisputed province. The most curious instance, perhaps, of quite recent years is the attempt of Edebohls, of New York, to apply surgery to the treatment of chronic Bright's disease. He calls his operation "Decapsulation of the Kidney," a term which sufficiently describes what it is. He has performed the operation in 51 cases, and though his results so far are not perhaps specially encouraging, they are certainly not less so than has frequently been the case with new operations that have eventually won a position of repute. If surgery continues to intrude into the domains of medicine in this manner the future outlook for the pure physician does not appear very happy; for the seller of patent medicines, the tabloid manufacturer, and the electrical quack already keep many patients from his door.

### Overcrowded Kensington.

It will probably come as somewhat of a surprise to anyone who has not chanced to glance through the overcrowding statistics of the Medical Officer for the County of London, to learn that as regards overcrowding Kensington occupies quite a high place. No fewer than 55 per cent. of its "houses" are tenements of less than five rooms, and in these live 73,225 persons out of its total population of 176,628. The term overcrowding in the sense in which it is used by the Medical Officer of Health of the London County Council applies to any tenement which being one of less than five rooms is made to provide shelter for more than two persons per room. The term applies to the conditions under which nearly 15 per cent. of the entire population of Kensington live, while nearly 22 per cent. of it live in tenements of two rooms and under. The least "overcrowded" districts of the Administrative County of London are Lewisham and Wandsworth. In the former less than 3 per cent. of the population, and in the latter less than 5 per cent., live under conditions of overcrowding. The worst of all is Finsbury with 35 per cent.

### What is an Apothecary?

The title apothecary is as likely to fall into desuetude as that of "barber-surgeon" has done, for though it still exists it is not regarded with favour by its legal possessors, and is being met with less and less frequently. An endeavour to revert to the title, for the sole use of those persons who, being dispensers of medicines, hold what is called "the certificate of an assistant apothecary," granted by either of the Apothecaries' Halls of London and Dublin, would appear to be the aim and object of a pamphlet now lying before us. No doubt a feeling exists among a section of qualified dispensers of medicine that they should be granted a legal designation to distinguish them from chemists and druggists. By usage the term apothecary has come to mean something more than a mere compounder of drugs and dispenser of medicine. Its original meaning in the Greek had reference to a storehouse. Chaucer applies it to a dealer in drugs and compounder of medicines:—

> "Ther was also a Doctour of Phisik,
> . . . . . .
> Ful redy hadde he his apotecaries,
> To sende him dragges and his letuaries."
> *The Prologue*, lines 412, 427–8.

In the sense understood by the Apothecaries' Company in the seventeenth century the term still has reference to a compounder of medicine, but in the early Victorian period an apothecary was something more, for he was permitted to charge, not only for medicines, but also for attendance upon patients, for whom he could prescribe; in fact, he could practise, with certain restrictions, as a medical man. In the course of the last century the examination for the L.S.A. was reconstructed, these restrictions were removed, and while retaining the title of apothecary the licentiate became a regular medical man, qualified to practise medicine, surgery, and midwifery, and having equal rights with other medical practitioners. In fact, at the present time, legally as well as actually, an "apothecary" is a physician and surgeon. It is curious to note that the writer of the brochure referred to, with respect to these changes, regards the attitude of the Apothecaries' Halls towards qualified dispensers as unjust. Are we to understand they are not content? "Seeing," he writes, "that Licentiates of the Halls no longer desire to be termed Apothecaries, I see no earthly reason why Graduates" (sic) "in Pharmacy, etc., of the Halls should not assume the title, or rather re adopt it, seeing that it is in abeyance." If his object be to obtain for qualified dispensers a suitable designation, which shall indicate that they are neither chemists nor druggists, we quite agree with him; but a title should be chosen which will not only distinguish dispensers from chemists and druggists, but, what is more important, which will also distinguish them from qualified medical men.

